# Recruitment and retention of fathers with young children in early childhood health intervention research: a systematic review and meta-analysis protocol

**DOI:** 10.1186/s13643-019-1215-1

**Published:** 2019-12-01

**Authors:** Elizabeth M. Keys, Jill M. Norris, Emily E. Cameron, Katherine S. Bright, Lianne M. Tomfohr-Madsen, Karen M. Benzies

**Affiliations:** 1 Faculty of Nursing, University of Calgary, PF2278, 2500 University Drive NW, Calgary, Alberta T2N 1 N4 Canada; 2 Department of Psychology, University of Calgary, Calgary, Alberta Canada; 3 Alberta Children’s Hospital Research Institute (ACHRI), Calgary, Alberta Canada; 4 Department of Pediatrics, Cumming School of Medicine, University of Calgary, Calgary, Alberta Canada; 5 Department of Community Health Sciences, Cumming School of Medicine, University of Calgary, Calgary, Alberta Canada

**Keywords:** Systematic review, Fathers, Infant, Father-child relations, Research subject recruitment, Intervention studies, Attrition

## Abstract

**Background:**

Fathers are under-represented in research and programs addressing early childhood health and development. Recruiting fathers into these interventions can be hampered for multiple reasons, including recruitment and retention strategies that are not tailored for fathers. The primary aim of this systematic review and meta-analysis is to determine the effectiveness of recruitment and retention strategies used to include fathers of children (from conception to age 36 months) in intervention studies. The secondary aim is to investigate study-level factors that may influence recruitment and retention.

**Methods:**

We will conduct searches for scholarly peer-reviewed randomized controlled trials, quasi-experimental studies, and pre-post studies that recruited fathers using the following databases: MEDLINE (Ovid), EMBASE (Ovid), PsycINFO (Ovid), and CINAHL. English-language articles will be eligible if they recruited self-identified fathers of children from conception to age 36 months for health-promoting interventions that target healthy parents and children. Two reviewers will independently screen titles/abstracts and full texts for inclusion, as well as grading methodological quality. Recruitment and retention proportions will be calculated for each study. Where possible, we will calculate pooled proportional effects with 95% confidence intervals using random-effects models and conduct a meta-regression to examine the impact of potential modifiers of recruitment and retention.

**Discussion:**

Findings from this review will help inform future intervention research with fathers to optimally recruit and retain participants. Identifying key factors should enable health researchers and program managers design and adapt interventions to increase the likelihood of increasing father engagement in early childhood health interventions. Researchers will be able to use this review to inform future research that addresses current evidence gaps for the recruitment and retention of fathers. This review will make recommendations for addressing key target areas to improve recruitment and retention of fathers in early childhood health research, ultimately leading to a body of evidence that captures the full potential of fathers for maximizing the health and wellbeing of their children.

**Systematic review registration:**

PROSPERO CRD42018081332.

## Background

Fathers contribute to the cognitive development, physical health, and social, emotional, and behavioral functioning of their children, over and above the contribution of mothers [1–8]. Fathers nurture, show affection, are responsive to their children’s needs, and offer distinct parenting practices and experiences that differ from mothers [3, 6, 9–11]. Despite this acknowledgment, mothers remain the focus of research about young children, with fathers continuing to be under-represented in both research and programs addressing early childhood health and development [12–15]. For example, in a recent systematic review of early childhood sensitivity and attachment interventions that included 743 participants within 4 studies, only 2 participants (< .01%) were male [16]. This dearth extends to observational research, with a systematic review of 667 studies noting only 17% of the total participants were fathers [17]. Further, Rominov and colleagues’ [18] systematic review found only 11 studies between 1994 and 2012 that examined father-focused mental health interventions. When compared to the maternal mental health intervention research described in several reviews of systematic reviews [19, 20], the notable difference between health research targeting mothers compared to fathers is reinforced, such that fathers are consistently a minority of the participant population.

Until the 1970s, fathers were rarely included in such research because societal norms and early theories of child development stressed the maternal role as primary caregiver with the belief that fathers had minimal direct influence on their children’s development and functioning [13, 14, 21]. However, fathers have increasingly become more involved in their children’s lives and care [4, 9, 21, 22]. Not only do fathers have a direct impact on their children’s growth and development, but the support they provide for mothers is essential to enhance child outcomes [4, 22]. In his review, Hoffman [22] concluded that “optimal support from the father can have a substantially positive impact on the mother’s capacity for optimal caregiving and interaction with her baby” (p. 34). Yet, when compared to mothers, relatively little is known about effective interventions to help fathers support their partner and their children’s development [23].

Recruiting fathers of children of any age to participate in research has proven challenging [24–27]. Potential obstacles to fathers’ participation in child health and parenting interventions include not asking fathers to participate [28], maternal gatekeeping [29], work commitments, lack of time, and perceived gendered parenting roles [30]. In research examining father engagement in domestic violence prevention, the accessibility of programs (i.e., timing and location of services, costs, stigmatizing program names, and childcare availability), staff attitudes and behaviors, program marketing, and program format (i.e., use of groups, literacy level of media, and duration) were found to be potential contributors to father engagement [31]. Panter-Brick and colleagues [23] identified design and delivery conditions inherent to many parenting interventions that act as barriers to father engagement. Many barriers are modifiable and could be mitigated by increased awareness and father-inclusive design. Moreover, designing interventions that do not artificially isolate and address only the father-child relationship or the parenting couple’s relationship could prove useful in creating an integrative approach whereby relational elements (fathering and parental/family relationships) are considered together [32]. Our experience in conducting observational studies suggests that many fathers of young children want to be involved in research [33–39]. In our intervention studies, fathers indicated that they were eager to learn about how to support their child’s early development [40–42]. Thus, while there is overall support for early childhood health research becoming more father inclusive, as well as suggested target areas to address for engaging fathers, the evidence base examining the effectiveness of specific recruitment and retention methods has not yet been systematically analyzed.

### Objectives

To support the ultimate goal of increasing the evidence base pertaining to interventions that help fathers support early childhood health, the objective of this review is to identify recruitment, retention, and research strategies to facilitate the participation of fathers of young children (from conception to age 36 months) in intervention research. The questions guiding this systematic review and meta-analysis are the following:

1. How effective are the recruitment and retention strategies used to include fathers (from conception to age 36 months) in intervention studies?
2. What are the potential study-level factors that influence recruitment and retention rates?

## Methods

### Design

We used the Preferred Reporting Items for Systematic reviews and Meta-analyses Protocols (PRISMA-P) statement [43] to guide this protocol (see Additional file 1) and have registered with PROSPERO CRD42018081332. We will document any amendments to the protocol with a rationale and report them with the final publication.

### Eligibility criteria

#### Participants

We will include studies of self-identified fathers of children from conception to age 36 months who participated in an intervention targeting medically and psychologically healthy parents and children. For this review, interventions that target medically or psychologically unwell individuals (i.e., mother, father, child) will be excluded. The rationale for this exclusion is that the underlying motivation of unwell individuals or family members of unwell individuals to engage in intervention research may be greater and/or specific based on the potential for additional treatment opportunities of an immediate concern. Thus, the review is limited to recruitment of fathers for health research that targets well individuals.

#### Interventions

We define an intervention as any educational or psychoeducational program that focuses on promoting health, wellbeing, or development of one or more family members, including the prevention of disease or health-harming behaviors. Interventions will include those targeted towards fathers, the family unit, mothers, or their child. Interventions for unwell individuals will be excluded, as per the participant inclusion criterion.

### Outcomes

#### Primary outcome

Similar to other reviews of recruitment in intervention research [44, 45], recruitment for fathers will be calculated as a proportion of the number of fathers enrolled in research projects at baseline out of the total number of fathers identified as eligible.

#### Secondary outcomes

Additional recruitment proportions will be calculated based on previously described approaches to assessing recruitment [46, 47] that use, when available, (a) the total number of potential eligible individuals (pool), (b) the number of invited individuals from the pool (invited), (c) individuals who respond to the invitation to participate (responders), and (d) participants who were assessed as eligible and enrolled in the study (started). Recruitment rates will be calculated using previously described approaches [46–48] that calculate a rate based on the number of participants who started participation at each recruitment site divided by the total recruitment period (defined as the time in months between the start and completion of recruitment). Similar to Walters et al. [48], we will include proportions of actual to target sample sizes.

We will calculate retention based on a previously described strategy [49], in which the number of participants (fathers) retained in research projects until the time point of primary assessment outcome was collected is compared to the total number of participants enrolled. For studies with follow-up beyond the primary outcome collection time point, we will evaluate how many fathers were retained at each follow-up contact points. To calculate attrition rates, we will calculate the number of fathers lost during follow-up out of duration between follow-up time points.

### Study type

Randomized controlled trials, quasi-experimental studies, and single group pre-post studies will be included in the review. Program evaluations that were not formalized intervention studies will be excluded. In cases of duplicate publications of the same sample, we will include the publication with the largest sample size. If sample sizes are consistent, the first published study from the sample will be included. We will exclude conference papers, dissertations, and other non-peer reviewed reports due to available resources for this review, the likelihood of these publication types having greater risk of unclear or high risk of bias. We will exclude reviews, as the primary focus of this review is on recruitment and retention methods used in primary research studies. Non-English publications will be excluded based on the study team’s inability to translate non-English languages. The effect of excluding unpublished works, dissertations, and non-English reports is anticipated to be small [50].

### Information sources and search strategy

MEDLINE (Ovid; 1946 to present), EMBASE (Ovid; 1974 to present), PsycINFO (Ovid; 1806 to present), and CINAHL (EBSCO; 1937 to present) databases will be searched. The search strategy was created with the assistance of an information scientist, and subject headings were modified to ensure they were database specific. The provisional MEDLINE search strategy is available in Table 1. In addition, we will conduct forward citation searches in Google Scholar, search the reference lists of included studies, and ask experts to identify further studies for inclusion. We will also hand search the reference lists of relevant systematic review articles. No filters will be used and no language restrictions will be placed. EndNote X8 will be used to manage the records, remove duplicates, and find full texts. We will re-run the searches immediately prior to the final analyses to retrieve any addition recently published articles for inclusion.

**Table 1:** Provisional search strategy

1	exp Fathers/	7877
2	father*.mp.	41857
3	dad.mp.	5999
4	dads.mp.	759
5	paternal.mp.	23160
6	or/1-5	65155
7	exp Education, Nonprofessional/ or exp Prenatal Education/	188637
8	psychoeducation*.mp.	4021
9	psycho-education*.mp.	1229
10	education*.tw,kf.	486444
11	intervention*.mp.	847036
12	program*.mp.	877690
13	or/7-12	2010750
14	exp postpartum period/	59206
15	exp Labor, Obstetric/	45665
16	exp Labor, Induced/	9107
17	exp pregnancy/	853992
18	exp Parturition/	14311
19	exp infant/	1088414
20	postpartum.mp.	61905
21	post-partum.mp.	11505
22	postnatal.mp.	99728
23	post-natal.mp.	6709
24	antenatal.mp.	31014
25	ante-natal.mp.	516
26	perinatal.mp.	67248
27	peri-natal.mp.	184
28	pregnan*.mp.	946625
29	birth*.mp.	334360
30	childbirth.mp.	18779
31	infancy.mp.	59520
32	infant.mp.	1136965
33	infants.mp.	244504
34	toddler*.mp.	9054
35	neonat*.mp.	266522
36	baby.mp.	34818
37	babies.mp.	34051
38	prenatal.mp.	157509
39	pre-natal.mp.	1202
40	labor.mp.	117454
41	labour.mp.	28432
42	newborn*.mp.	724924
43	or/14-42	2306627
44	6 and 13 and 43	4681
45	prepartum.mp.	2024
46	pre-partum.mp.	313
47	43 or 45 or 46	2306701
48	6 and 13 and 47	4681

Database(s): Ovid MEDLINE(R) Epub Ahead of Print, In-Process & Other Non-Indexed Citations, Ovid MEDLINE(R) Daily and Ovid MEDLINE(R) 1946 to present

### Study selection

Prior to screening titles and abstracts, we will conduct training and a calibration exercise with the review team to refine the screening tool in Microsoft Excel. Titles/abstracts will be independently screened by pairs of two reviewers. Disagreements will be resolved by a third reviewer. Full texts of potential studies will then be obtained and screened in the same manner with disagreements discussed as a review team.

### Data items and data extraction

Table 2 details the data items to be extracted from the full texts, which are informed by the Template for Intervention Description and Replication (TIDieR) [51] and Transparent Reporting of Evaluations with Nonrandomized Designs (TREND) checklists [52]. Extracted data will include study characteristics, participants, intervention characteristics (including length and intensity), participant flow, assignment methods, recruitment methods, and retention methods. Data on recruitment and retention methods will be categorized and coded to better allow for assessment of how these methods impact recruitment and retention. We will use a templated data extraction tool in Microsoft Excel. Once the team completes a calibration exercise with the extraction tool, one reviewer will extract study data with a second reviewer verifying the extracted data. Discrepancies in extracted data will be resolved through discussion. Missing data or clarifications regarding ambiguous data will be requested by email from study authors.

**Table 2:** Data extraction categories for eligible studies

Category	Data extracted
Study characteristics	First author, year, country, study design
Participant	Eligibility criteria (including different levels in recruitment/sampling plan), demographics, data collection setting
Intervention characteristics	Content, delivery method, unit of delivery, deliverer, setting, exposure quantity and duration, time span, effect size of intervention
Assignment method	Unit of assignment Method used to assign units to study conditions, including details of any restriction Inclusion of aspects employed to help minimize potential bias comparison condition induced due to non-randomization
Participant flow	Flow of participants: enrollment, assignment, allocation and intervention exposure, baseline and outcome measures, follow-up
Recruitment	Method of recruitment, sampling method, recruitment setting
Retention	Method to increase compliance or adherence

### Study recruitment reporting quality

The quality of recruitment reporting will be assessed using the grading scheme developed by Foster et al. [47], in which 2 independent reviewers will score 5 criteria related to the reporting of recruitment as a binary score, with scores of 0 used for criteria not reported or inadequately reported and a score of 1 allocated for criteria that are adequately described. Studies scoring 4 or 5 will be considered high quality for reporting recruitment, while studies scoring less than or equal to 3 will be considered low quality for reporting recruitment.

### Risk of bias in individual studies

Studies will be included regardless of methodological quality. We will use the Cochrane risk of bias tool [53] to rate selection, performance, detection, attrition, reporting, and other biases in each study as either high, unclear, or low risk of bias. Two reviewers will independently assess all studies for quality, and disagreements will be resolved through discussion.

### Data synthesis

Synthesis of the data will be conducted according to the Cochrane guidance [54]. Should the data permit, a meta-analysis of the recruitment and retention proportions will be conducted using Comprehensive Meta-Analysis (CMA) software, version 3.0. Heterogeneity of the studies’ recruitment and retention proportions will be assessed by the non-parametric Cochran’s *Q* test, which assesses variance between studies and study populations. We will calculate the *I*^2^ index to evaluate the proportion of heterogeneity between studies. If heterogeneity is present, random-effects models (as opposed to fixed-effect models) will be used as they are a more appropriate computational approach under conditions of heterogeneity given they are less likely to reject the null hypothesis. Further, random-effects models are more robust to large variations in sample sizes [55]. Recruitment and retention will be calculated as a proportional effect size with confidence intervals reported based on a 95% criterion. These proportions will be computed first as a proportional effect size and then transformed into logit units using CMA, which allows for more variability in the data than the bound proportional effect sizes [56, 57]. We will also conduct a sensitivity analysis by sequentially removing one study at a time and reanalyzing the dataset to determine the impact of any given single study. In conjunction with the quality analysis previously mentioned, this procedure allows for the inclusion of methodologically flawed studies if they meet this criterion. We will use a ± 5% range for acceptable, such that if one study affects the meta-estimate by more than ± 5%, it will be flagged for further consideration regarding inclusion in the meta-estimate. In addition, we will stratify the meta-analyses by high and low risk level of individual study bias, based on “high” ratings of bias for more than three of the six domains of bias examined by the Cochrane risk of bias tool and including a high level of risk for attrition bias. We will also stratify the meta-analyses by high and low recruitment reporting quality, based on a score of > 4 or < 3 on the quality of recruitment reporting grading scheme, respectively. Providing that there is sufficient information, we will also carry out meta-regression using logit units to explore potential modifiers of recruitment and retention rates, such as (a) study characteristics, (b) recruitment methods, (c) retention methods, (d) father and family characteristics, and (e) intervention characteristics. In the case that the included studies are too heterogeneous for meta-analysis, we will aggregate findings using narrative synthesis [58].

### Meta-bias

Publication bias will be assessed through visual inspection of a funnel plot as well as statistical tests (e.g., Egger’s regression intercept, Begg and Mazumdar’s rank correlation, and Orwin’s fail-safe *N*) [59–61].

### Confidence in cumulative evidence

GRADE (Grades of Recommendation, Assessment, Development and Evaluation) [62] will be used to aid in evaluating the overall quality of the evidence in five domains: limitation of study design and execution, inconsistency, indirectness, imprecision, and publication bias. The overall quality of the evidence—rated as high, moderate, low, or very low—will be presented in a summary of findings table for each of the primary and secondary outcomes.

## Discussion

To our knowledge, this protocol describes the first systematic review and meta-analyses that specifically examines the recruitment and retention rates of fathers in early childhood health intervention research. Previous reviews have described the lack of engagement of fathers in parenting interventions for children of all ages [23], domestic violence prevention [31], and obesity treatment and prevention research [63]. This systematic review will describe actual recruitment and retention strategies of early childhood health research, as well as the resulting recruitment and retention rates, including meta-analyses of participant and study characteristic subgroups. As such, we will be able to compare actual recruitment and retention rates with perceived facilitators and barriers [30, 64].

Our target knowledge users for our review findings are early childhood health intervention researchers and program managers. Accordingly, our dissemination strategies will target venues that researchers and program managers typically engage in to facilitate dissemination of findings. These venues include presentation of review results at local and national or international meetings, as well as publication in a peer-reviewed open-access journal. In addition, the research team will create and distribute a summary infographic, a promising visual knowledge translation tool [65], via social media, which has been reported to be used by health researchers and clinicians for professional purposes [66].

Limitations to the review may include limited data that may preclude the ability to run meta-analyses on all potential sub-groups of participant and study characteristics. Additionally, there may be limited descriptions of recruitment and retention strategies in the articles under review. Further, while articles may include fathers, data may only be presented on the couple and not disaggregated by mothers and fathers. Further limitations are related to the inclusion/exclusion criteria of reviewing only English-language articles, which may reduce generalizability to non-English speaking populations. Similarly, the inclusion of only peer-reviewed literature excludes government reports, dissertations, conference papers, and reviews thereby potentially increasing publication bias in our review and limiting grass-roots or community-based recruitment and retention strategies that may be used to target smaller or marginalized groups of fathers.

Findings from this review could help researchers design and develop more effective and evidence-based recruitment and retention strategies that enable and encourage father-inclusive early childhood health research. Managers of early childhood health programs who are interested in adopting a more father-inclusive approach may also be interested in using the review findings to consider how study-level factors may compare to program elements and promote or hinder father enrollment and retention. Identifying key facilitators and evidence gaps related to recruitment and retention of fathers in early childhood health intervention research should contribute to the overall body of evidence that aims to harness the potential of fathers in improving the health and wellbeing of their children.

## Supplementary information

**Additional file 1.** PRISMA-P checklist.

## Data Availability

The datasets created and analyzed during this review will be available from the corresponding author upon reasonable request. We will also deposit our datasets in an open access repository, Open Science Framework (https://osf.io/), developed and maintained by the Centre for Open Science.

## Abbreviations

GRADE: Grades of Recommendation, Assessment, Development and Evaluation
PRISMA-P: Preferred Reporting Items for Systematic reviews and Meta-analyses Protocols
TIDieR: Template for Intervention Description and Replication
TREND: Transparent Reporting of Evaluations with Nonrandomized Designs
